# The experience of Stigma on Recovery Among Parents with Serious Mental Illness: A Systematic Review

**DOI:** 10.1192/j.eurpsy.2025.1858

**Published:** 2025-08-26

**Authors:** M. G. Pijnenborg, E. sportel, I. Hasson-Ohayon, I. Bogers, C. Prins

**Affiliations:** 1 university of Groningen, Groningen; 2 GGZ Drenthe, Assen, Netherlands; 3 bar-ilan university, tel aviv, Israel

## Abstract

**Introduction:**

The parental role is often central to the recovery process. For the 70% of people with Serious Mental Illnesses (SMI) who are or will become parents, the parental role is often central to their recovery process. Although a growing number of studies focuses on recovery in parents with SMI, little is known about the experienced influence of stigma on recovery within this specific group.

**Objectives:**

This systematic review aims to provide a deeper understanding of the interaction between various types of stigma, the parental role, and the recovery process in parents with SMI.

**Methods:**

A systematic search was conducted in four online databases and the reference lists of included articles. Primary research studies of any design that included stigma- and recovery-related experiences among parents with SMI were included. Stigma-related experiences were categorized into six distinct types. The findings of included studies were inductively coded. Reflexive Thematic Analysis (RTA) was applied for the data-analysis.

**Results:**

A total of 25 studies were included, all of which identified various types of stigma related to the parental role. The data analysis of the interaction between the parental role, stigma, and recovery resulted in the conceptualization of six themes: (1) Loneliness and isolation; (2) Struggling with parental identity; (3) Protecting the children; (4) Not having the same rights and chances; (5) Lost in the system and (6) Overcoming stigma.

**Conclusions:**

Parents with SMI experience stigmatization of their condition as well as stigmatization of their parental role. Stigmatization of the parental role can have a profound impact on their recovery process, since it limits or even restricts parents from fulfilling their parental role. In order to protect their parental role and family life, parents are hesitant to disclose their SMI and ask for help. As a result, parents and their families are hindered in receiving the needed (psychiatric) support. Since the parental role is central to the recovery process for parents with SMI, it is crucial for Mental Health professionals to pay specific attention to the stigma- and recovery-related factors associated with this role.

**Disclosure of Interest:**

None Declared

